# Coordinated Decision Control of Lane-Change and Car-Following for Intelligent Vehicle Based on Time Series Prediction and Deep Reinforcement Learning

**DOI:** 10.3390/s24020403

**Published:** 2024-01-09

**Authors:** Kun Zhang, Tonglin Pu, Qianxi Zhang, Zhigen Nie

**Affiliations:** Faculty of Transportation Engineering, Kunming University of Science and Technology, Kunming 650500, China; putonglin@stu.kust.edu.cn (T.P.); 20212206076@stu.kust.edu.cn (Q.Z.); niezhigen@kust.edu.cn (Z.N.)

**Keywords:** intelligent vehicles, time series prediction, deep reinforcement learning, lane-change and car-following, condition identification, trajectory planning, coordinated control

## Abstract

Adaptive cruise control and autonomous lane-change systems represent pivotal advancements in intelligent vehicle technology. To enhance the operational efficiency of intelligent vehicles in combined lane-change and car-following scenarios, we propose a coordinated decision control model based on hierarchical time series prediction and deep reinforcement learning under the influence of multiple surrounding vehicles. Firstly, we analyze the lane-change behavior and establish boundary conditions for safe lane-change, and divide the lane-change trajectory planning problem into longitudinal velocity planning and lateral trajectory planning. LSTM network is introduced to predict the driving states of surrounding vehicles in multi-step time series, combining D3QN algorithm to make decisions on lane-change behavior. Secondly, based on the following state between the ego vehicle and the leader vehicle in the initial lane, as well as the relationship between the initial distance and the expected distance with the leader vehicle in the target lane, with the primary objective of maximizing driving efficiency, longitudinal velocity is planned based on driving conditions recognition. The lateral trajectory and conditions recognition are then planned using the GA-LSTM-BP algorithm. In contrast to conventional adaptive cruise control systems, the DDPG algorithm serves as the lower-level control model for car-following, enabling continuous velocity control. The proposed model is subsequently simulated and validated using the NGSIM dataset and a lane-change scenarios dataset. The results demonstrate that the algorithm facilitates intelligent vehicle lane-change and car-following coordinated control while ensuring safety and stability during lane-changes. Comparative analysis with other decision control models reveals a notable 17.58% increase in driving velocity, underscoring the algorithm’s effectiveness in improving driving efficiency.

## 1. Introduction

As artificial intelligence technology advances, intelligent vehicles have emerged as a prominent application [1]. The rapid evolution of autonomous driving technology in recent decades is driven by its expansive potential applications [2]. Crafting a well-considered lane-change trajectory involves weighing various factors including safety, economy, and comfort [3]. Moreover, it necessitates accounting for the kinematic and dynamic constraints inherent to the vehicle [4]. Lane-changing trajectory planning stands out as a key area of research within the transportation domain [5].

Driving behavior decision-making is a pivotal technology in the realm of intelligent vehicles, with lane-changing decision-making serving as a fundamental requirement for ensuring the safe transition of vehicles between lanes. Current methodologies for lane-change decision-making can be broadly classified into traditional-rule-based models [6,7,8,9] and machine-learning-based models [10,11,12,13,14,15]. Reference [16] proposed the Gipps model, and formulated decision rules based on the necessity, propensity, and safety considerations of lane-change. Subsequent research further refined lane-change behaviors into categories such as free, collaborative, and forced lane-change [17,18,19]. However, reference [20] contended that traditional-rule-based models fall short in accurately representing the myriad factors influencing drivers during lane-change decision-making, and proposed a lane-change decision-making model based on Random Forest (RF), which had good prediction accuracy.

Reference [21] analyzed the relationship between feature variables and lane-change decision, and proposed a lane-change decision model based on Gradient Boosting Decision Tree (GBDT), which introduced new feature variables to improve the prediction performance of the decision model. Reference [22] selected feature variables from the physical characteristics of vehicles, interaction perception and road structure for different lane-change conditions to establish a decision model. Reference [23] used reinforcement learning to solve the accurate decision parameters during lane-change, which improved the safety performance and could mimic real driving behavior in lane-change scenarios. A large number of scholars have conducted research on lane-change trajectory planning. Reference [24] dynamically planned the lane-change time and increased the comfort constraints of lane change to improve the quintic polynomial planning algorithm. Moreover, they introduced the concept of a lane-change transit position and proposed a double quintuple polynomial algorithm. This algorithm effectively ensured collision avoidance with the lead vehicle. However, an oversight in their study was the neglect of considering the influence of the state of vehicles in the target lanes on the selection of the transit position. Reference [25] introduced a lane-change trajectory planning method designed for dynamic environments, which computed the optimal time interval for horizontal planning, diminished the array of candidate trajectories, and notably enhanced real-time trajectory planning performance. However, it exclusively addressed horizontal trajectory, neglecting longitudinal trajectory planning. Reference [26] delineated the selection of an optimal reference trajectory through a comprehensive evaluation system grounded in the vehicle’s state. This trajectory was fitted using a segmented quintic polynomial, enhancing the vehicle’s obstacle avoidance capabilities. However, a significant disparity existed between the reference trajectory and the optimized trajectory.

Mere consideration of lane-change behavior for decision-making and trajectory planning falls short of addressing the practical requirements of driving. Moreover, there exists a significant correlation between lane-change and car-following, which are among the most prevalent traffic behaviors [27,28,29]. Reference [30] discussed the control requirements of the ACC system for both accelerating to follow the vehicle in front and executing lane-change. The proposed cooperative control strategy guaranteed satisfactory longitudinal following performance and lateral stability. In reference [31], the implementation of Model Predictive Control (MPC) algorithm enabled dual target tracking and safe lane-change, effectively resolving conflicts between ACC and lane changing behaviors, thereby enhancing longitudinal comfort. Reference [32] introduced a designed ACC multi-mode switching strategy based on the cumulative degree of speed dissatisfaction. This strategy achieved adaptive cruise control with lane-change functionality, leading to improved driving efficiency.

The construction of lane-change scenarios in the aforementioned models are simplistic and lack a comprehensive consideration of the impact of surrounding vehicles on the safe lane-change maneuvers of the subject vehicle. Moreover, these models lack the integration of decision-making and trajectory planning. Additionally, the collaborative decision control involving adaptive cruise and lane-change behavior warrants more in-depth investigation. Given the shortcomings of the above research, this paper introduces a coordinated decision-control of lane-change and car-following for intelligent vehicles based on time series prediction and deep reinforcement learning. The contributions of this paper are as follows:

(1) A hierarchical lane-change and car-following coordinated decision-making control model aimed at improving driving efficiency is established, which divides the lane-change trajectory planning problem into longitudinal velocity planning and lateral trajectory planning, and the trajectory is planned based on the driving condition identification.
(2) Multi-step time series prediction information is introduced to realize the prediction of the future driving state of the surrounding vehicles, which provides the basis for lane-change decision-making and trajectory planning.
(3) A three-layer safety guarantee mechanism of a decision-making layer, planning layer and control layer is constructed to ensure the safety of the whole lane-change and car-following process.
(4) The lane-change data in the NGSIM dataset are extracted to construct the training scenario, and the lane-change scenarios dataset is established to improve the authenticity and complexity of the training environment, and to verify the effectiveness of the model.

## 2. Analysis of Lane-Change Behavior

**Figure 1:**
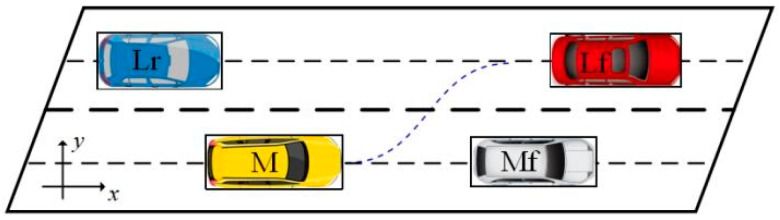
Schematic diagram of lane-change scenario.

The motion state of surrounding vehicles influences the lane-change behavior of ego vehicle, and the decision to change lane must entail the consideration of various traffic factors. Taking change lane to the left as an example, a lane-change scenario with multi-vehicle influences containing Ego M, the original lane leader Mf, the target lane leader Lf, and the target lane following Lr is established as shown in Figure 1. In addition, we consider the overtaking to the right and curved lane-change as dangerous driving behaviors, and we have not accounted for them.

According to the vehicle kinematics, the position of the centroid of each vehicle during the lane-change process from the starting moment $t_{s}$ to the ending moment $t_{f}$, can be expressed as:

(1) $$\left\{\begin{array}{c} x_{i}(t)=\int_{t_{s}}^{t_{f}}[v_{i}(t)cos\varphi_{i}(t)]dt\\ y_{i}(t)=\int_{t_{s}}^{t_{f}}[v_{i}(t)sin\varphi_{i}(t)]dt \end{array}\right.$$

where $v_{i}(t)$ is the longitudinal velocity, $\varphi_{i}(t)$ is the vehicle heading angle, *i* = M, Mf, Lf, Lr.

In the vehicle coordinate system with the centroid of the vehicle as the origin, the potential collision points of the vehicle in the lane-change process are the left front end point of the vehicle body $P_{1}$, the right front end point $P_{2}$, and the left rear end point $P_{3}$. According to the analysis of the potential collision points in the process of lane-change, combined with the driving state of the leader and following vehicles in the target lane, taking the left lane-change as an example, establish the constraints for safe lane-change within the duration $t\in [t_{s},t_{f}]$, and the calculation formula is shown in (2).

(2) $$\left\{\begin{array}{c} x_{M_{P_{1}}}(t)\leq x_{{Lf}_{P_{4}}}(t)+S_{M,Lf}(t_{s})-S_{M,Lf}^{safe}\;\;\;\;\\ y_{M_{P_{1}}}(t)\leq y_{{Lf}_{P_{4}}}(t)\;\;\;\;\;\;\;\;\;\;\;\;\;\;\;\;\;\;\;\;\;\;\;\;\;\;\;\;\;\;\;\;\;\;\;\;\;\;\;\; \end{array}\right.\;\left\{\begin{array}{c} x_{M_{P_{2}}}(t)\leq x_{{Mf}_{P_{3}}}(t)+S_{M,Mf}(t_{s})-S_{M,Mf}^{safe}\;\;\\ y_{M_{P_{2}}}(t)\leq y_{{Mf}_{P_{3}}}(t)\;\;\;\;\;\;\;\;\;\;\;\;\;\;\;\;\;\;\;\;\;\;\;\;\;\;\;\;\;\;\;\;\;\;\;\;\;\;\;\; \end{array}\right.\;\left\{\begin{array}{c} x_{M_{P_{3}}}(t)\geq x_{{Lr}_{P_{2}}}(t)-S_{M,Lr}(t_{s})+S_{M,Lr}^{safe}\;\;\;\;\;\\ y_{M_{P_{3}}}(t)\geq y_{{Lr}_{P_{2}}}(t)\;\;\;\;\;\;\;\;\;\;\;\;\;\;\;\;\;\;\;\;\;\;\;\;\;\;\;\;\;\;\;\;\;\;\;\;\;\;\;\; \end{array}\right.$$

where $S_{i,j}(t_{s})$ is the longitudinal distance between the following car and the preceding car at the moment, $S_{i,j}^{safe}$ is the longitudinal reserved safety distance.

The purpose of reserving the longitudinal safety distance with the leader and following vehicles during the lane-change process is to ensure enough active collision avoidance distance when the leader and following vehicles have acceleration and deceleration behavior. Since the vehicle is in the following state with the leader vehicles before and after lane change, this paper uses the following vehicle safety distance model [33], as shown in (3).

(3) $$S_{i,j}^{safe}=v_{m}t_{r}+\frac{{v_{m}}^{2}}{2a_{m}}+L-\frac{{v_{m}}^{2}}{2a_{f}}$$

where $v_{m}$ is the initial velocity of following vehicle before braking, $t_{r}$ is the brake coordination time, *L* is the safe distance between leader vehicle and following vehicle after stopping, $a_{m}$ is the maximum braking deceleration of following vehicle, $a_{f}$ is the maximum braking deceleration of leader vehicle.

**Figure 2:**
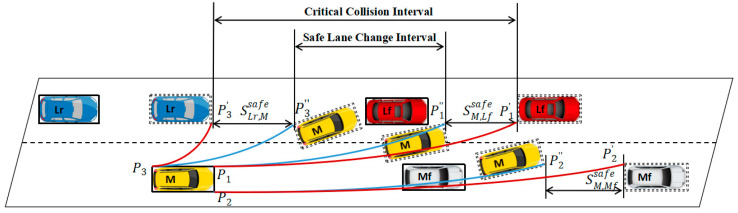
Safe lane change interval.

As shown in Figure 2, circular arc $\hat{P_{3}P_{3}^{\prime }}$, $\hat{P_{1}P_{1}^{\prime }}$ and $\hat{P_{2}P_{2}^{\prime }}$ constitute the critical collision interval, in this area, lane-change vehicles will not collide with surrounding vehicles. After introducing the safe distance maintenance model between vehicles, circular arc $\hat{P_{3}P_{3}^{\prime \prime }}$, $\hat{P_{1}P_{1}^{\prime \prime }}$ and circular arc $\hat{P_{2}P_{2}^{\prime \prime }}$ constitute the safe lane-change interval, which ensures that there will be no collision when the leader and following vehicles have acceleration and deceleration behavior.

## 3. Overview of the Framework

**Figure 3:**
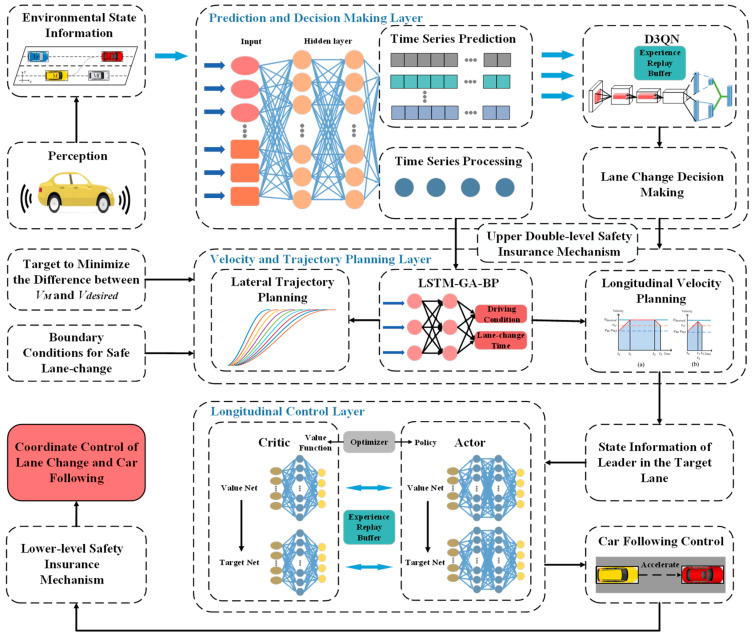
Overview of the framework.

In order to pursue higher driving efficiency, this paper establishes a coordinated decision-control model for lane-change and car-following based on hierarchical time series prediction and deep reinforcement learning, which consists of a decision-making layer, a trajectory planning layer and a lower control module. The framework proposed in this paper is shown in Figure 3.

The decision-making layer comprises the time series prediction and lane-change decision module. The Long Short-Term Memory (LSTM) network undertakes the processing of time series information pertaining to both the surrounding vehicles and the self-vehicle. Concurrently, it engages in a multi-step time series prediction of the state information associated with the surrounding vehicles. The resulting time series prediction information is then fed into the Dueling Double Deep Q Network (D3QN) algorithm. Subsequently, the D3QN model produces the decision-making outcome for the lane-change behavior, drawing upon the current moment’s state and time series prediction information. Upon receiving the lane-change command, the planning layer assumes responsibility for lateral and longitudinal planning based on Genetic Algorithm (GA) improved LSTM-BP neural network (GA-LSTM-BP), which realizes the driving condition identification and predicting the time of lane-change. Different longitudinal velocity planning according to different driving conditions, based on the time series prediction information and boundary condition for safe lane-change, allows us to determine the safety by simulating the lane-change. And the lower control module is the Deep Deterministic Policy Gradient (DDPG) algorithm, which is responsible for the realization of the car-following action under different driving conditions. The assurance of lane-change and car-following safety is achieved across three levels: decision-making level, trajectory planning level, and lower control level.

## 4. Lane-Change Decision-Making Model

### 4.1. Time Series Prediction Module

The safety constraints during vehicle lane-change are influenced by both the lateral and longitudinal positions of surrounding vehicles. Given the inherent uncertainty in the driving behaviors of these surrounding vehicles, precise prediction of their future motions becomes paramount. This precision is essential for determining optimal safe lane-change intervals and subsequently planning trajectories that ensure safe and effective lane-change.

The time series prediction module must comprehend the vehicle’s driving pattern using acquired state information and anticipate its future driving state, aligning with the demands of multi-step timing prediction. While neural networks yield favorable results in predicting nonlinear system problems, straightforward feed-forward models like BP neural networks and radial basis neural networks lack the necessary memory function for handling time-dependent data sequences. Consequently, these models are ill-suited for predicting the driving state of a vehicle based on time series data.

In the multi-step time series prediction problem, compared to NARX (Nonlinear Auto Regressive with exogenous inputs) network, LSTM (Long Short-Term Memory) network can learn more complex time patterns and regularities [34] for more accurate and reliable multi-step time series prediction. The inputs to the LSTM network contain the historical trajectory information of the surrounding vehicles, which can be expressed as:

(4) $${Fh}_{i}=[x_{i}(t),v_{i}(t),a_{i}(t)],t=T-T_{h},\cdots ,T-1,T$$

where *i* = Mf. Lf, Lr, $x_{i}(t)$ are the historical positions, $v_{i}(t)$ are the historical velocities, $a_{i}(t)$ are the historical accelerations, and $T_{h}$ is the historical time domain, reflecting the length of input information.

Without considering the lane-change behavior of surrounding vehicles, the output can be expressed as:

(5) $${Fp}_{i}=[X_{i}(t),V_{i}(t),A_{i}(t)],t=T,T+1,\cdots ,T+T_{p}$$

where $X_{i}(t)$ are the predicted longitudinal positions of surrounding vehicles, $V_{i}(t)$ are the predicted longitudinal velocities, $A_{i}(t)$ are the predicted longitudinal accelerations, and $T_{p}$ is the predicted time domain, reflecting the prediction length of multi-step time series prediction. If the historical time domain is set too small, it cannot capture enough historical state information, leading to a lack of comprehensive understanding of the current state in the model, resulting in erroneous decisions. Conversely, it will make the model overly focus on historical information, increase fitting, and lead to overfitting and low training efficiency. After testing, the $T_{h}$ is set to 5 s, and the $T_{p}$ is 4 s. The time series prediction information will be fed into the decision-making module, trajectory planning module and lower level control module.

To guarantee adherence of the planned trajectory to the actual driving state and to ensure optimal traffic efficiency, safety, and comfort, the duration of lane-change is restricted. This is under the assumption of favorable road surface conditions and the vehicle being subject to constraints in both lateral and longitudinal dynamics. Each parameter is detailed in Table 1.

### 4.2. Lane-Change Decision-Making Based on Time Series Prediction and Deep Reinforcement Learning

The D3QN algorithm is developed based on Deep Q-Network (DQN) and Double Deep Q-Network (DDQN) in Deep Reinforcement Learning. Compared with the latter two, the D3QN algorithm is improved in terms of the model structure, the estimation of the value function, and the improvement of the policy to improve the stability and the performance of the algorithm. D3QN introduces three neural networks: the main network, the target network and the averaging network, where the main network is used to update the values and select the actions, the target network is used to compute the target values and assist in the training, and the averaging network is used to average the two networks in order to further reduce the error and improve the performance. The main network is divided into two neural networks for estimating the state value function and the dominance function, respectively. The state value function is used to estimate the expected cumulative reward obtained in the current state, while the dominance function is used to estimate the additional benefit of performing a specific action relative to the average, and the value can be viewed as a weighted sum of the state value function and the dominance function:

(6) $$Q(s,a;\theta ,\alpha )=V(s;\theta ,\beta )+(A(s,a;\theta ,\alpha )-\frac{1}{\left|A\right|}\sum_{a^{\prime }}A(s,a^{\prime };\theta ,\alpha ))$$

where *s* is states, *a* is action, α, θ and β are parameters of different neural networks, $\frac{1}{\left|A\right|}\sum_{a^{\prime }}A(s,a^{\prime };\theta ,\alpha )$ is the sum of the average dominance functions of all possible actions $a^{\prime }$. The core idea of the deep Q-network algorithm lies in approximating the optimal *Q*-value function $Q^{*}(s,a)$, by means of a neural network, which can be expressed as:

(7) $$Q(s,a;\theta )\approx Q^{*}(s,a)$$

where Q(s,a;θ) is the predicted *Q* value for a given state *s* and action *a*.

The reward function is established from the aspects of lane-change benefit and safety. In the coordinated control of lane-change and car-following model, the purpose of ego vehicle is to obtain higher driving velocity. Ego vehicle maintains the following relationship with the leader vehicle before and after the lane-change, and the maximum velocity that the ego vehicle can achieve in the initial lane is $v_{Mf}$, and the maximum velocity in the target lane is $v_{Lf}$. The premise of the vehicle lane-change is to ensure the safety, not to collide with other vehicles, the whole process of lane-change needs to maintain a safe distance from other vehicles. The composite reward function is:

(8) $$reward=g[k_{1}(\frac{v_{desired}}{v_{Mf}}-1,\frac{v_{Lf}}{v_{Mf}}-1)+k_{2}S_{i,j}]$$

where $v_{desired}$ is the desired velocity of ego vehicle, $k_{1}$, $k_{2}$ are the weighting coefficients of each weighting, which were set to 0.9 and 0.6 after testing; $S_{i,j}$ is the distance between the ego vehicle and each of the surrounding vehicles; and g is the compensation function, which aims to reduce the order of magnitude difference of the different values of each reward.

(9) $$state=\{s_{M},s_{i},{Fp}_{i}\}$$

where $s_{M}=(v_{M},a_{M})$ is the state information of ego vehicle, $s_{i}=(exist,x_{i},v_{i},a_{i})$ means the state information of surrounding vehicles, exist = [0, 1] represents whether there are leader or following vehicles in the lane, with 0 indicating no presence and 1 indicating presence, when no vehicle exists in the lane, the state information of the vehicle i is set to: following vehicle $x_{i}=-\infty$, leader vehicle $x_{i}=+\infty$, $v_{i}=v_{M}$.

In the structured road scenario, the model proposed in this paper does not consider the right lane-change behavior, and the D3QN algorithm outputs binary classification results with the action space defined as:

(10) action={1,0}

where 1 represents a lane-change to left, 0 represents a lane-keeping behavior.

## 5. Lateral and Longitudinal Trajectory Planning Based on Driving Condition Recognition

### 5.1. Lateral Trajectory Planning and Driving Condition Recognition

At the level of lateral trajectory planning, a quintic polynomial curve is employed to plan the lateral trajectory. The pivotal factor influencing comfort during lateral displacement is the maximum lateral acceleration. The magnitude of the lateral acceleration is only related to the lane-change time, and the maximum lateral acceleration determines the minimum lane-change time. As lane-change time increases, lateral acceleration decreases, correlating with improved comfort. However, it comes at the expense of reduced traffic efficiency. Addressing diverse driving conditions and recognizing the vehicle’s dynamic state as a time series process, a hybrid LSTM-BP neural network model, incorporating a genetic algorithm, is formulated. The LSTM neural network algorithm captures sequential relationships preceding and following lane-changing data, while the subsequent application of the BP neural network excavates nonlinear connections between each variable, lane-change time $t_{f}$, and driving conditions recognition.

**Figure 4:**
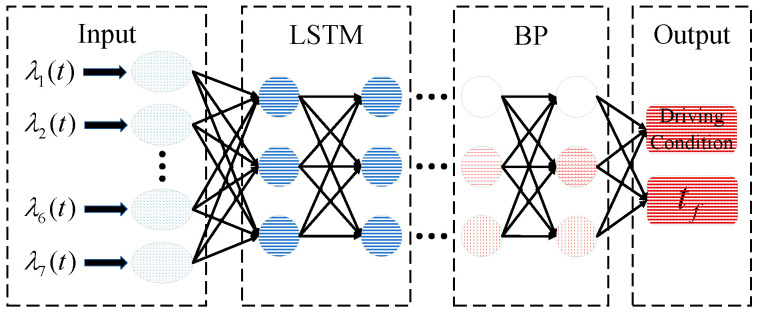
GA-LSTM-BP structure diagram.

Upon receiving the lane-change decision instruction output from the D3QN model, the GA-LSTM-BP model processes a set of time series data $\lambda_{i}$ spanning 4 s prior to the initiation of the lane-change. These data include critical parameters such as the velocity of the ego vehicle, the relative velocity, and the relative distance between the ego vehicle and the leader vehicle in the initial lane. Similarly, it considers the relative velocity and distance concerning both the target lane’s lead vehicle and the following vehicle. The GA-LSTM-BP model produces outputs indicating the lane-change time and the recognition results for the driving conditions throughout the lane-change process. A structural depiction of the network is presented in Figure 4.

### 5.2. Longitudinal Velocity Planning Considering the Car-Follow Characterization

When the ego vehicle is not following the leader vehicle in the initial lane, the ego vehicle will travel at the desired velocity. On the contrary, when it is following the leader, it will travel at the velocity of the leader, the velocity of leader is greater than the desired, there will be no benefit to change lane, then the velocity relationship of each vehicle in the scenarios is set to:

(11) $${v_{desired}>v}_{Lf}>v_{Mf}$$

According to whether the ego vehicle and the leader vehicle in the initial lane are in the following state or not, and the relationship between the initial distance and the desired distance between the ego vehicle and the leader vehicle in the target lane, this paper describes the lane-change and car-following scenarios in six driving conditions, as shown in the Table 2.

The primary objective of vehicular following is twofold: firstly, to maintain the desired distance from the leader vehicle, and secondly, to facilitate velocity synchronization, ensuring a stable trailing condition. The essence of both lateral and longitudinal planning lies in the achievement of lane-change and the optimization of driving efficiency.

In contrast, the conventional adaptive cruise control system adjusts velocity only after attaining the desired distance from the preceding vehicle, focusing solely on velocity following. Regrettably, this approach falls short in meeting the demands of a concurrent working scenario involving both lane-change and car-following. Recognizing this limitation, longitudinal velocity planning, which takes into account the following characteristics, aims to concurrently achieve the desired distance and enable velocity following:

(12) $$\left\{\begin{array}{c} ∆S=S-S_{desired}\\ ∆v=v_{M}-v_{Lf}\;\;\;\; \end{array}\right.$$

where ∆S is the following distance error, ∆v is the relative velocity, and $S_{desired}$ is the desired distance from the leader vehicle, which can be expressed as:

(13) $$S_{desired}=v_{M}t_{h}+d_{0}\geq S_{i,j}^{safe}$$

where $t_{h}$ is time headway, $d_{0}$ is a constant distance.

Upon obtaining the driving condition recognition results from the GA-LSTM-BP model, diverse longitudinal velocity planning strategies are implemented based on distinct driving conditions.

**Figure 5:**
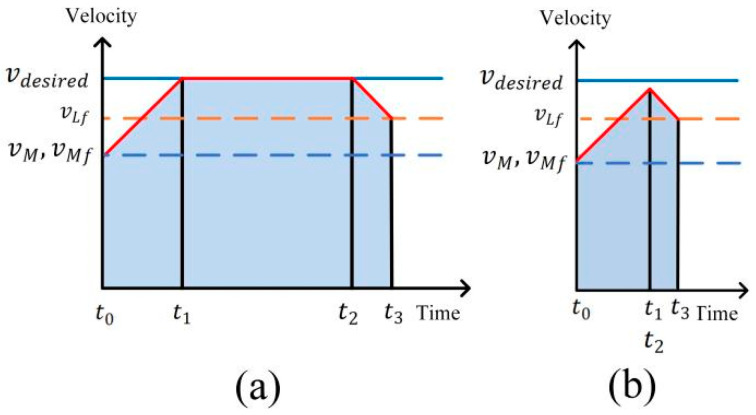
Velocity planning for Condition 1: (**a**) large distance; (**b**) shorter distance.

Driving Condition 1: In instances where the initial distance between the ego vehicle and the leader vehicle in the target lane surpasses the desired distance, a three-phase approach is adopted by the ego vehicle to ensure optimal driving efficiency while establishing and maintaining a following relationship with the front vehicle. These phases involve accelerating or decelerating with maximum acceleration to reach the desired velocity $t_{a}\in [t_{0},t_{1}]$, maintain a constant velocity $t_{u}\in [t_{1},t_{2}]$, subsequently decelerating to match the velocity of the leader vehicle as the distance decreases $t_{d}\in [t_{2},t_{3}]$. At the point when the ego vehicle attains the desired distance, it aligns its velocity with the leader vehicle, as depicted in Figure 5a. Conversely, when the initial distance with the front vehicle is shorter, the ego vehicle is constrained to accelerate to a specific velocity and promptly decelerate, as illustrated in Figure 5b.

In Figure 5a, the ego vehicle is able to achieve the desired velocity:

(14) $$t_{a}=(v_{desired}-v_{M})/a_{x_{max}}\;t_{d}=(v_{Lf}-v_{desired})/a_{x_{min}}\;S_{a}=({v_{desired}}^{2}-{v_{M}}^{2})/2{/a}_{x_{max}}\;S_{d}=({v_{Lf}}^{2}-{v_{desired}}^{2})/2{/a}_{x_{min}}$$

where $S_{a}$ is the longitudinal displacement in time period $t_{a}$, and $S_{d}$ is the longitudinal displacement in time period $t_{d}$.

In order to achieve velocity following while reaching the desired distance from the leader vehicle, when the ego vehicle reaches the reaction distance $S_{r}$ from the leader vehicle, the ego vehicle starts to decelerate and achieves velocity following:

(15) $$S_{r}=S_{desired}+S_{d}$$

The longitudinal displacement to maintain the desired velocity can be calculated as:

(16) $$S_{u}=t_{u}v_{desired}$$

In Figure 5b, the ego vehicle can only slow down immediately after reaching a certain velocity $v_{c}$, $v_{c}$, $t_{a}$ and $t_{d}$ can be solved by the system of equations:

(17) $$\left\{\begin{array}{c} t_{a}=(v_{c}-v_{M})/a_{x_{max}}\;\;\;\;\;\;\;\;\;\;\;\;\;\;\;\;\;\;\;\;\;\;\;\;\;\;\;\;\;\;\;\;\;\;\;\;\;\;\;\;\;\;\;\;\;\;\;\;\;\;\;\;\;\;\;\;\;\;\;\;\;\;\;\;\;\;\;\;\;\;\;\;\;\;\;\;\;\;\;\;\;\;\;\;\\ t_{d}=(v_{c}-v_{Lf})/a_{x_{max}}\;\;\;\;\;\;\;\;\;\;\;\;\;\;\;\;\;\;\;\;\;\;\;\;\;\;\;\;\;\;\;\;\;\;\;\;\;\;\;\;\;\;\;\;\;\;\;\;\;\;\;\;\;\;\;\;\;\;\;\;\;\;\;\;\;\;\;\;\;\;\;\;\;\;\;\;\;\;\;\;\;\;\;\\ S_{M,Lf}(t_{S})+v_{Lf}\times (t_{a}+t_{d})-S_{r}=(v_{c}+v_{M})\times \frac{t_{a}}{2}+(v_{c}+v_{Lf})\times \frac{t_{d}}{2} \end{array}\right.$$

**Figure 6:**
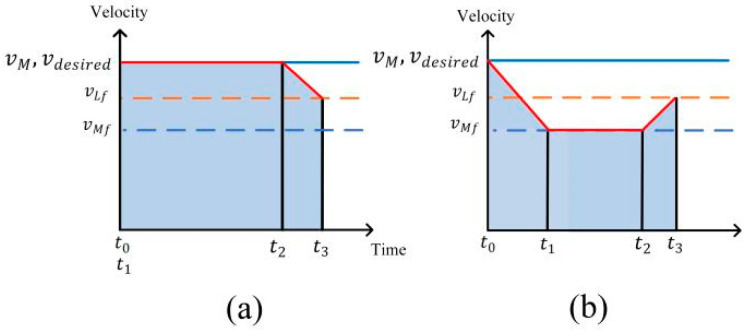
Velocity planning: (**a**) Condition 2; (**b**) Condition 3.

Driving Condition 2: When the initial distance between the ego vehicle and the leader vehicle in the target lane is larger than the desired distance, and the autonomous vehicle is not in the following state with the leader vehicle in the initial lane, the ego vehicle will change lane in advance if the safety conditions of lane-change are satisfied, and directly realize the following with the leader vehicle in the target lane, as shown in Figure 6a.

Driving Condition 3: If the safety conditions for lane-change cannot be met, the ego vehicle continues to travel, follows the leader vehicle in the initial lane first, and then carries out the operation of changing lane to follow the leader vehicle in the target lane, as shown in Figure 6b.

**Figure 7:**
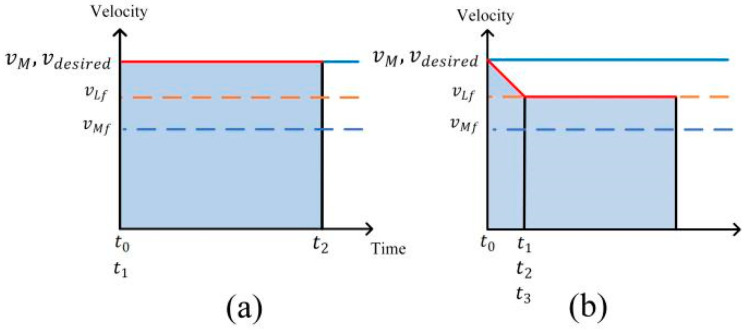
Velocity planning: (**a**) Condition 4; (**b**) Condition 5.

Driving Condition 4: When the initial distance between the ego vehicle and the leader vehicle in the target lane is less than the desired distance, and the ego vehicle is not in the following state with the leader vehicle in the initial lane, if the distance between the ego vehicle and the leader vehicle in the initial lane is large, the ego vehicle will change lane after overtaking the leader vehicle in the target lane in accordance with the desired velocity in the initial lane as shown in Figure 7a.

Driving Condition 5: When the distance to the leader vehicle in the initial lane is not enough to overtake the vehicle and then change lane, the ego vehicle needs to adjust its velocity first to widen the distance to the leader vehicle in the target lane, and then follow it as shown in Figure 7b.

**Figure 8:**
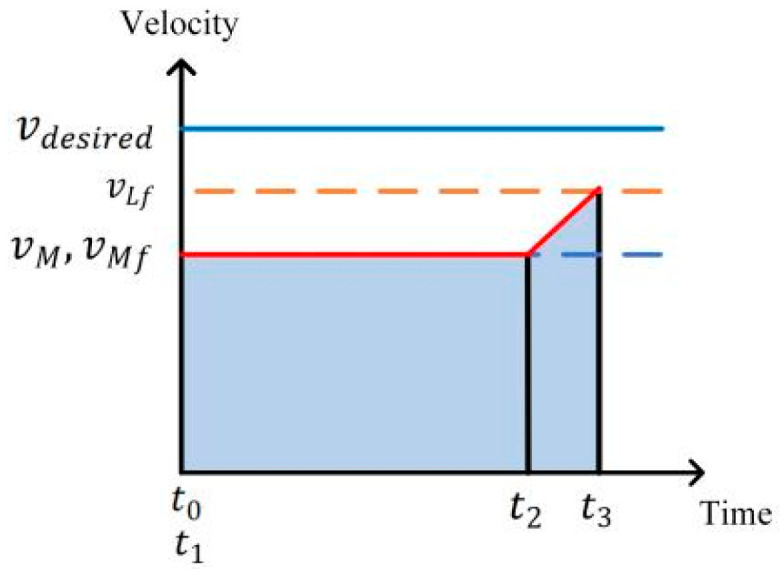
Velocity planning for Condition 6.

Condition 6: When the initial distance between the ego vehicle and the leader vehicle in the target lane is less than the desired distance, the ego vehicle needs to adjust its velocity to open up the distance first, and then follow the vehicle. When $v_{Lf}>v_{M}$, in order to ensure the driving efficiency, the ego vehicle first maintains a constant velocity and then accelerates to realize the following, as shown in Figure 8, and the time of driving at a constant velocity can be calculated as:

(18) $$\;\;\;\;\;\;\;\;\;t_{u}=-\frac{v_{Lf}t_{d}-S_{M,Lf}(t_{s})-S_{r}}{v_{desired}-v_{Lf}}$$

**Figure 9:**
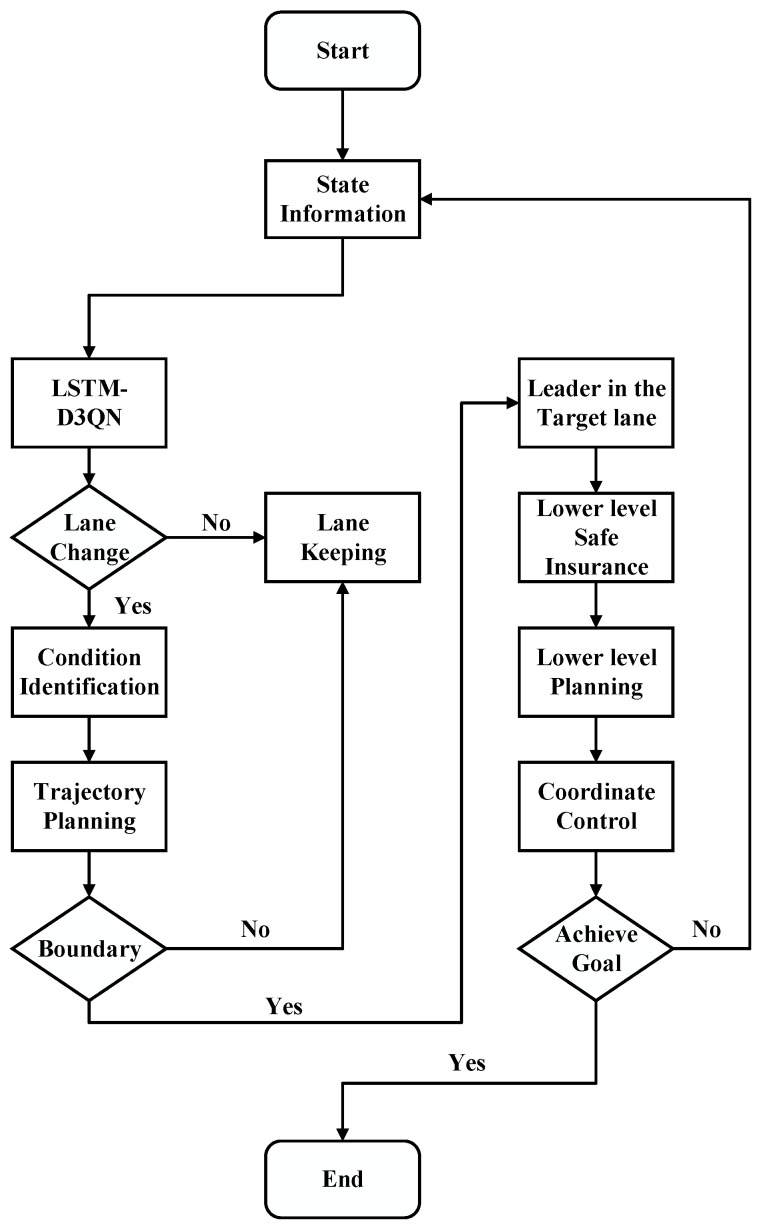
Technical route of the coordinated decision control model.

After the lateral and longitudinal trajectory planning, the planning safety is verified by simulating the lane-change by combining the timing prediction information and the safety boundary for lane-change. If the planned trajectory does not meet the boundary, lane keeping behavior is still adopted. The whole implementation method of the lane-change and car-following model is shown in Figure 9.

## 6. DDPG-Based Lower Level Control Model

The DDPG algorithm is an extension of the deep Q-network by applying it to the problem of continuous action space. It solves this problem by simultaneously training an Actor network, which learns approximate policy functions and is able to output continuous action values directly, and a Critic network, which is used to evaluate the Q-value function of state–action pairs. A key component of DDPG is the Experience Replay Buffer (ERB), which is used to store previous experience samples and randomly sample from them for training. This can effectively solve the problem of correlation between data and increase the efficiency of sample utilization.

The design of the reward function directly affects the effect of the deep reinforcement learning algorithm, and the reward function is set as follows:

(19) $$reward=\alpha_{1}{\left(v_{ref}(t)-v_{M}(t)\right)}^{2}+\alpha_{2}a_{M}^{2}(t-1)$$

where $\alpha_{1}$, $\alpha_{2}$ are the weight coefficients, which are tested and take the values of −0.1 and 1, respectively, $v_{ref}(t)$ is the reference velocity at the current moment, and $a_{M}(t-1)$ is the acceleration of the ego vehicle at the previous moment. According to the content of longitudinal velocity planning in Section 5, when the reaction distance $S_{r}$ is not reached, $v_{ref}$ is set according to different driving conditions, and when the reaction distance is reached, $v_{ref}=v_{Lf}$.

The action space is defined as:

(20) $$action=\{a_{M}\in [-3,2]\}$$

The state space is defined as:

(21) $$state=\{v_{M},v_{Mf},v_{Lf},S_{r},a_{Lf}\}$$

The deep reinforcement learning-based model realizes safe car-following in a dynamic environment, and together with the upper decision-making module and the velocity planning module, it ensures the safety of the combined lane-change and car-following conditions.

## 7. Simulation Verification

**Figure 10:**
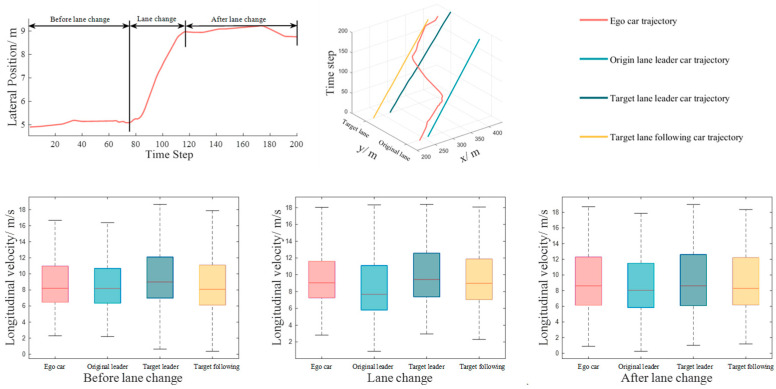
NGSIM dataset Descriptions.

NGSIM US-101 and I-80 vehicle trajectory data are widely used in the study of vehicle lane-change behavior [35]. This dataset is obtained through video analysis of the vehicle’s position, velocity, acceleration and other state information. Since there is a certain degree of noise, this paper uses the sliding average filtering method [36] to process the raw data, after which the free lane-change data in the dataset are extracted, according to [37], and an analysis of the lane-change decision-making process is carried out to process the lane-change data and to extract the state information of the vehicles around the ego vehicle. Based on the constraint of lane-change time, this paper extracts 247 vehicle trajectory datasets with a duration of 20 s. The whole trajectory is divided into three phases: before lane-change, after lane-change and lane-change, and the average velocities of the ego vehicles in the dataset in the three phases are 8.61 m/s, 9.55 m/s, and 9.12 m/s, and that of the leader vehicles in the target lane are 9.58 m/s, 10.08 m/s, and 9.06 m/s, and the average velocities of the leader vehicle in the initial lane in the first phase is 8.53 m/s. As shown in Figure 10. It can be seen that in the real lane-change scenarios, the ego vehicle tends to gain higher velocity and change lane, which is in line with the lane-change gain of the lane-change decision module in this paper. Meanwhile, 500 lane keeping data are extracted and used for training to validate the time series prediction, lane-change decision-making, and lateral trajectory planning modules.

### 7.1. Lane-Change Decision-Making Model Training Results

In the driving scenario proposed in this paper, the lane-change decision is a binary classification problem, and a control group is set up to verify the effectiveness of the proposed time series prediction and deep reinforcement learning decision-making model. In addition, a test control group is set up with (1) the support vector machine model proposed in the reference [8], and (2) DDQN with the same settings of reward function, state space and action space as the algorithm proposed in this paper. In addition, in order to verify the effect of time series prediction on the lane-change decision, each group of the above experiments is divided into a module with time series prediction and a module without it, and the training results are shown in Table 3.

Evaluation of machine learning models’ training effectiveness is needed from the quantitative indicators perspective. The Accuracy, True Positive Rate (TPR), and True Negative Rate (TNR) are introduced to evaluate the training effectiveness of the model. The Accuracy of a model reflects its ability to correctly classify samples and is calculated as the ratio of correctly classified samples to the total number of samples. Similarly, the TPR measures the rate at which positive instances are correctly predicted to be positive, while the TNR measures the rate at which negative instances are correctly predicted to be negative. Since the decision to change lane depends on other vehicles in the traffic environment, misclassifying non-lane-changes as lane-changes poses greater risks than misclassifying lane-changes as non-lane-changes. Therefore, a good lane change decision model should have high ACC and TNR.

Overall, different classification decision models have improved all three quantitative metrics after adding LSTM time series prediction information, indicating that the prediction of the future driving state of surrounding vehicles has a positive impact on lane-change decision. The method based on the combination of time series prediction and deep reinforcement learning substantially improves the prediction accuracy compared to the traditional machine learning model, and the proposed lane-change decision model achieves the best training results, with 94.30% correct prediction rate and 95.10% true negative class rate, which is able to make safe and accurate lane-change decisions.

### 7.2. Validation of Lateral Trajectory Planning

**Figure 11:**
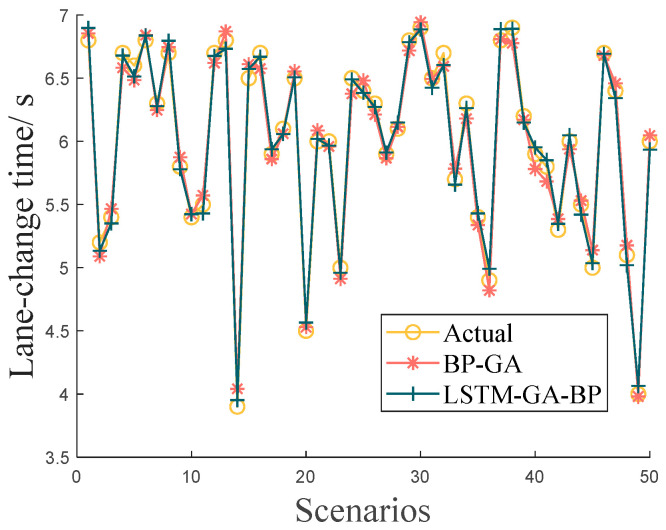
Prediction results of GA-LSTM-BP neural work.

The lane-change data in the NGSIM dataset are extracted to train and validate the trajectory planning module, and a comparison of the 50 sets of prediction results for the lane-change time of the LSTM-GA-BP neural network and GA-BP neural is shown in Figure 11. Table 4 shows the comparison results of the overall data.

It can be seen that the LSTM-GA-BP neural network has higher prediction accuracy, the average error and the root mean square error are smaller, the model can meet the prediction requirements and has better stability, and it can be used for the accurate prediction of lane-change time.

### 7.3. Car-Following Model Validation

**Figure 12:**
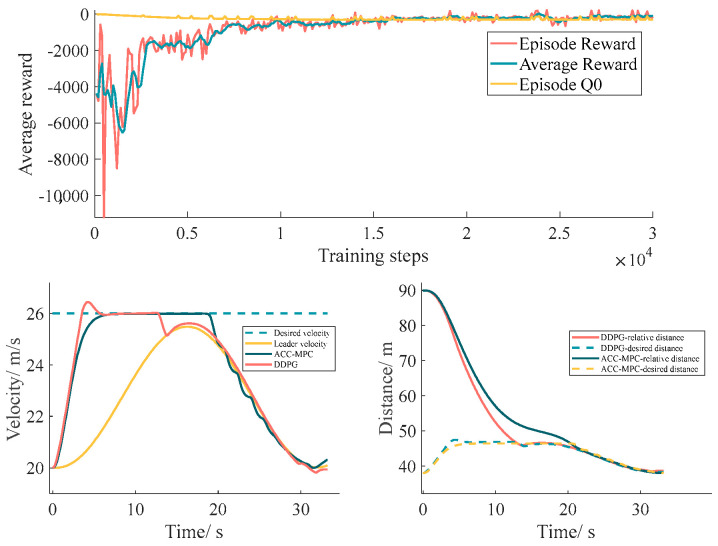
Car-following comparison results.

In the single lane environment, the DDPG heeling model is trained to compare the traditional ACC-MPC. The training process and the training results in the set scenario are shown in Figure 12. It can be seen that the DDPG-based adaptive cruise control can realize more stable following control in the case of the velocity change.

### 7.4. Overall Model Validation

The NGSIM dataset was used for the experimental scenarios, and the performances of different lane-change and car-following models were compared. The comparison results are shown in Table 5.

It can be seen that in real lane-change scenarios, SVM as a decision model has collision risk, which is related to its lower accuracy in predicting lane-change behavior. MOBIL-ACC also has a certain collision risk. The three-layer security mechanism proposed in this article performs well in terms of safety. Compared with the original NGSIM data, all models have improved driving efficiency. When combined with various decision models, DDPG has a smaller velocity improvement compared to ACC. The lane-change decision model based on the cumulative degree of velocity dissatisfaction (CD) has a smaller improvement in driving efficiency. The lane-change and car-following model proposed in this article achieved the maximum acceleration and velocity improvement while ensuring safety, with a velocity increase of 17.58%.

**Figure 13:**
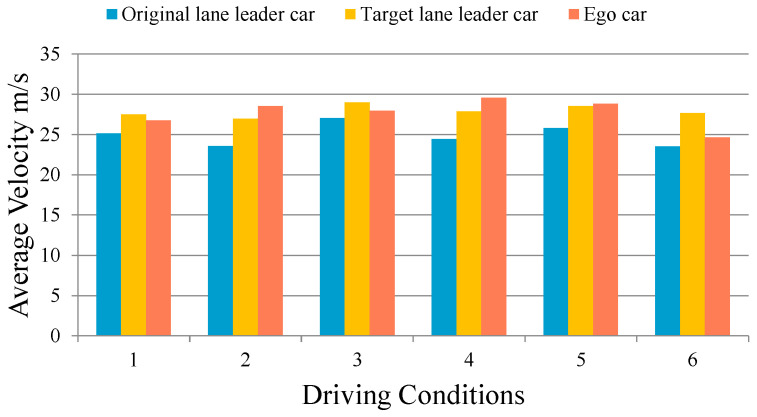
Lane-change scenarios simulation results.

In the NGSIM scenarios, the vehicle velocities are relatively low, and the number of samples that meet the lane-change and car-following scenarios is relatively small. In order to verify the adaptability of the proposed model in high-velocity scenarios and also verify the comprehensive performance of the six driving conditions mentioned in Section 5 above, a lane-change scenarios dataset was established. The duration of a single lane-change scenario is 20 s, and the velocities of surrounding vehicles in the scenario all range between 20–30 m/s. The expected velocity of the ego vehicle is 30 m/s. Based on the six driving conditions determined in the Table 2, the initial position, initial velocity, acceleration, acceleration start time, and acceleration duration of surrounding vehicles are randomly combined to simulate different traffic scenarios. After the acceleration duration ends, the vehicles simulate different traffic scenarios in a uniform manner. They are then driven at high velocity, with 100 sets of lane changing scenarios set for each operating condition. The simulation results are shown in the Figure 13.

It can be seen that compared to the leader vehicle in the initial lane, the proposed model can improve driving efficiency in different driving conditions. Among them, in driving conditions 2 and 4, the scenario type is one where the ego vehicle is not following the leader vehicle in the initial lane, which significantly improves driving velocity by changing lane in advance and after overtaking. It can be seen that the lateral and longitudinal trajectory planning based on driving conditions recognition is beneficial for improving driving efficiency in different scenarios. The proposed model is suitable for high-velocity scenarios.

## 8. Conclusions

This paper establishes a hierarchical decision control model based on time series prediction and deep reinforcement learning to achieve lane-change and car-following coordinated control of intelligent vehicles. The effectiveness of the proposed model was verified by extracting the NGSIM lane-change dataset and establishing a lane-change scene dataset. The results showed that the three-layer safety guarantee mechanism ensured safety, while increasing driving velocity by 17.58%. There are also shortcomings in this article. The accuracy of predicting the state information of surrounding vehicles needs to be improved. In addition, the driving conditions recognition module cannot fully cover the real driving conditions, and the poor quality of the training sample data in the NGSIM dataset has an impact on model training. In the future, we will consider collaborative control of intelligent connected vehicles in heterogeneous environments and develop decision control models for vehicles in extreme environments.

## Figures and Tables

**Table 1:** Kinetic constraints.

Parameter	Symbols	Value Range
Lane-change time	$t_{f}$	[3 s, 7 s]
Longitudinal velocity	$v_{M}$	[8 m/s, 34 m/s]
Longitudinal acceleration	$a_{M}$	[−3 m/s^2^, 2 m/s^2^]
Lateral acceleration	${\ddot{y}}_{M}$	[−2.4 m/s^2^, 2.4 m/s^2^]

**Table 2:** Driving conditions.

Conditions	Relationship To The Leader Vehicle In The Initial Lane	Relationship to the leader Vehicle in the Target Lane
1	following	Initial distance > desired distance
2	Not following	Initial distance > desired distance
3		
4	Not following	Initial distance < desired distance
5		
6	following	Initial distance < desired distance

**Table 3:** Decision-making comparison results.

Decision Model	LSTM+D3QN	D3QN	LSTM+DDQN	DDQN	LSTM+SVM	SVM
TPR	89.37%	88.64%	87.48%	84.90%	81.27%	80.98%
TNR	95.10%	93.79%	92.30%	91.90%	87.96%	83.03%
Accuracy	94.30%	93.27%	91.93%	90.08%	83.20%	81.60%

**Table 4:** Prediction comparison results.

	Maximum Error	Average Absolute Error	Root Mean Square Error
GA-BP	1.68 s	0.77	0.88
GA-LSTM-BP	1.24 s	0.48	0.56

**Table 5:** Model comparison results.

Lane Change Model	NGSIM	SVM	SVM	MOBIL	MOBIL	CD	D3QN
Car Following Model	NGSIM	ACC	DDPG	ACC	DDPG	ACC	DDPG
Collisions	0	12	10	4	0	0	0
Average Acceleration (m/s^2^)	0.17	0.35	0.37	0.44	0.51	0.23	0.54
Average Velocity (m/s)	9.09	9.31	9.25	10.36	10.49	9.78	10.68

## Data Availability

Data are contained within the article.
